# A case report and literature review on imaging manifestations of immature teratoma in fetal oral cavity with concurrent intracranial abnormalities

**DOI:** 10.3389/fonc.2026.1738161

**Published:** 2026-05-08

**Authors:** Jieting Fu, Qiaosheng Jiang, Chen Sun, Jiangfeng Pan

**Affiliations:** Department of Radiology, Jinhua Central Hospital, Jinhua, China

**Keywords:** case report, fetus, imaging, immature teratoma, intracranial abnormalities, oral cavity

## Abstract

**Objective:**

To explore the imaging characteristics of immature teratoma in fetal oral cavity and enhance the diagnostic and differential diagnostic ability for this condition.

**Methods:**

The clinical, pathological, and imaging features of a case of immature teratoma occurring in a fetus were analyzed. A systematic literature review was conducted, and prior cases were summarized in a comparative table to identify patterns and knowledge gaps.

**Results:**

Imaging manifestations of fetal oral cavity immature teratoma included: (1) A large solid-cystic mass protruding from the fetal oropharynx, closely related to the maxilla and skull base; (2) CT revealed heterogeneous density within the tumor, with a predominantly cystic appearance and punctate calcifications within the solid component—a feature more commonly associated with immature teratomas; (3) MRI demonstrated an irregular solid-cystic mass in the lesion’s oropharyngeal region, with low signal intensity on T1WI, internal areas of mixed signal intensity, high signal intensity on T2WI, and restricted diffusion (high DWI signal) within the solid components, corresponding to immature neuroepithelial elements on histopathology. Pathological examination confirmed a Grade III immature teratoma with concurrent right temporal arachnoid cyst and meningocele.

**Conclusion:**

Immature teratoma occurring in the fetal oral cavity is a rare condition. Multimodal imaging with systematic description of tumor location, composition, calcification, and mass effect enables accurate prenatal diagnosis and differentiation from other oral masses. Concurrent intracranial abnormalities, though uncommon, warrant thorough evaluation. Understanding its clinical and imaging features can improve the level of diagnostic proficiency for this disease.

## 1 Introduction

Teratomas originate from abnormal proliferation of germ cells and consist of tissues derived from all three germ layers. They commonly occur in the midline axial organs (1). In adults, teratomas often arise in the ovaries, while in fetuses, they frequently occur in the sacrococcygeal region (2). The occurrence of a teratoma in the fetal oral cavity, involving the maxilla and skull base, and concurrent intracranial abnormalities, is extremely rare. Due to the complex composition of teratomas, their imaging manifestations are diverse and challenging to diagnose. Prenatal diagnosis relies primarily on ultrasound examination. However, compared to ultrasound, MRI provides more comprehensive visual information, superior tissue density display, and can reveal the presence of associated malformations. It serves as an important complementary diagnostic tool to ultrasound. Fetal oral cavity teratomas are associated with high morbidity and mortality during the perinatal period, highlighting the importance of early diagnosis.

The novelty of this case lies in three aspects: (1) the direct imaging-pathology correlation established through multimodal imaging (prenatal MRI, postnatal CT) and histopathology in a Grade III immature teratoma; (2) the identification of concurrent intracranial abnormalities (arachnoid cyst and meningocele), which have not been previously reported in association with oral teratomas; and (3) the systematic imaging description that enables clear differentiation of immature from mature teratomas based on calcifications, invasive bone destruction, and restricted diffusion on MRI. These features collectively provide a valuable reference for radiologists and perinatologists encountering similar complex cases.

This study discusses the clinical manifestations, imaging features, diagnosis, and differential diagnosis of immature teratomas occurring in the fetal oral cavity based on relevant literature, aiming to enhance radiologists’ understanding of this condition.

## 2 Materials and methods

### 2.1 Case information

**Figure 1:**
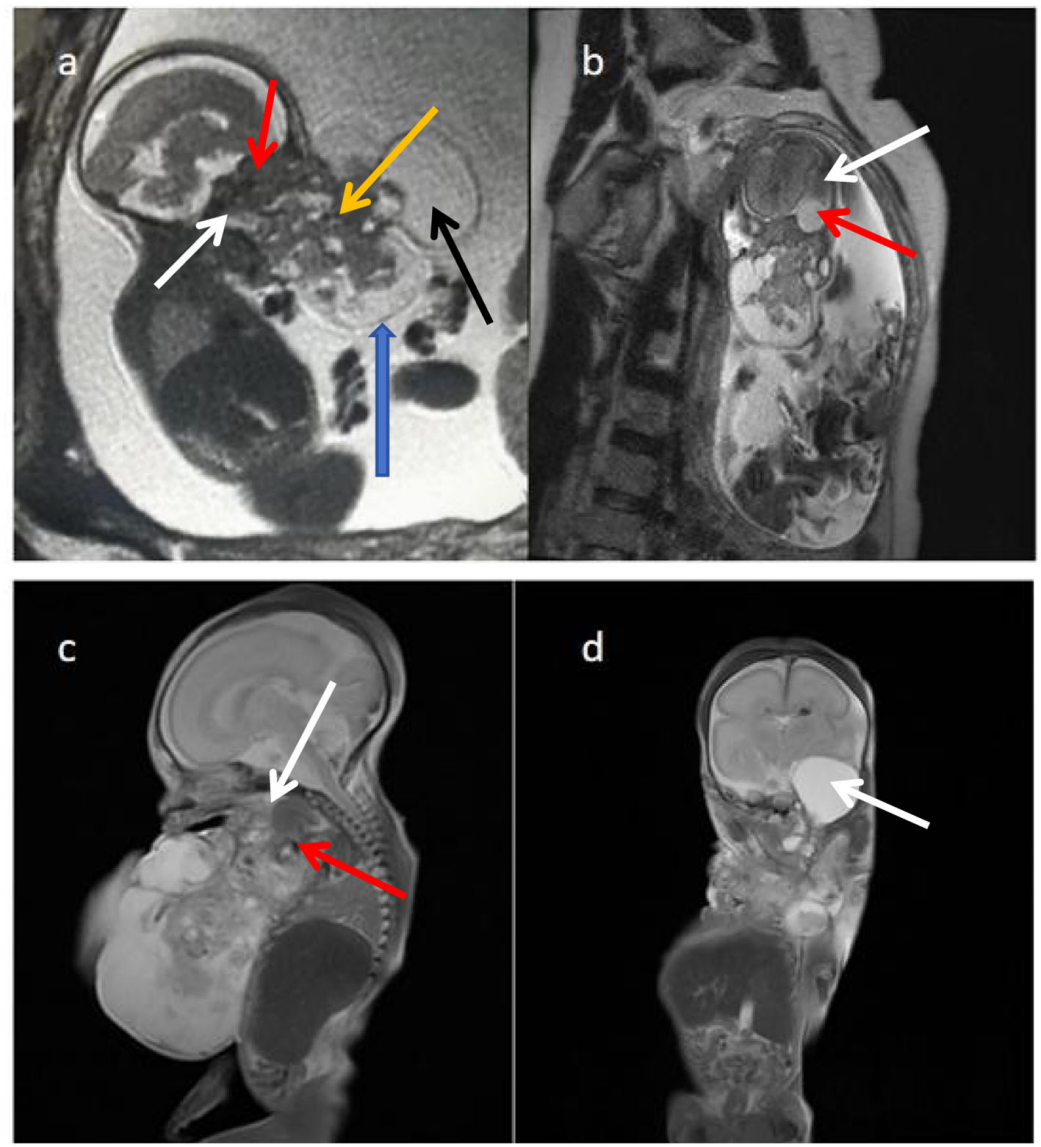
Prenatal and postnatal MRI findings of fetal oral immature teratoma with intracranial abnormality. **(a)** Prenatal sagittal T2-weighted MRI demonstrates a solid-cystic mass (blue arrows) measuring 6.1 × 6.1 × 6.8 cm arising from the oropharynx with broad attachment to the palate and skull base (red arrows). The solid component (S, orange arrows) shows intermediate signal intensity, while the cystic component (C, black arrows) exhibits markedly high T2 signal. The mass causes severe oropharyngeal compression (white arrows), correlating with the risk of upper airway obstruction. **(b)** Prenatal coronal T2-weighted MRI reveals a well-defined hyperintense lesion (red arrows) in the right temporal lobe, measuring 17 × 17 mm, compatible with an arachnoid cyst. Mass effect on the adjacent temporal lobe is evident (white arrows). The coexistence of an intracranial cystic lesion with an oral teratoma raises consideration of a developmental field defect involving midline structures. **(c)** Postnatal sagittal T2-weighted MRI confirms the broad-based attachment of the residual mass to the skull base and mandible (white arrows), with associated bony erosion (red arrows). **(d)** Postnatal coronal MRI shows a left temporal meningocele (arrow), an additional intracranial finding that further supports the presence of concurrent neurodevelopmental anomalies. S, solid component; C, cystic component.

Patient: Female, 45 years old. Chief complaint: Amenorrhea for 23+ weeks, fetal abnormality detected for 3 days. Medical history: The patient’s last menstrual period was on August 26, 2022, with an estimated due date of June 3, 2023. Menstrual cycles were regular. Irregular prenatal check-ups and no nuchal translucency (NT) examination in early pregnancy. On February 5, 2023, our hospital’s ultrasonography showed heterogeneous echoes in the fetal facial area, possibly originating from the oral cavity (measuring approximately 66 × 50 × 69 mm protruding externally with heterogeneous echoes). On February 6, 2023, our hospital’s fetal magnetic resonance imaging (MRI) (**Figure 1A**) suggested abnormal signals in the fetal oropharyngeal region, possibly originating from the oral cavity, considering a teratoma, and right temporal arachnoid cyst. On February 8, 2023, our hospital’s prenatal diagnostic ultrasound indicated a gestational age of 23 weeks. Mass in the fetal oral cavity and cystic echoes in the intracranial temporal region: tumor (teratoma)? and associated arachnoid cyst? (Limited visualization of the fetal lip, a heterogeneous echo measuring approximately 61 × 52 × 74 mm protruding externally, closely related to the intracranial cystic echo, suggesting a connection; a cystic echo with a size of approximately 23 × 22 mm visible in the right intracranial temporal region). No significant medical history, no family history of genetic diseases, congenital defects, or hereditary conditions.

Obstetric history: 2-0-1-1. Gave birth to a healthy male infant in 1999, who died from drowning at the age of 3. Gave birth to a healthy female infant in 2008. One induced abortion.

Laboratory examinations: No abnormalities found.

After understanding the condition, the patient and family requested termination of pregnancy. They agreed to undergo prenatal diagnostic procedures, induction of labor, fetal computed tomography (CT) and MRI examinations, and fetal autopsy.

### 2.2 Methods

Specimens were fixed in 10% neutral buffered formalin, dissected, and sampled for routine dehydration, paraffin embedding, sectioning at 4μm thickness, hematoxylin and eosin (HE) staining, and microscopic observation.

## 3 Results

### 3.1 Surgical procedure

The patient received antispasmodic and sedative treatment. Labor induction was performed using mifepristone and misoprostol, resulting in the delivery of a stillborn female infant (**Figure 1**) weighing approximately 970g and measuring approximately 30cm in length. A large tumor tissue measuring about 16cm×15cm×8cm protruding externally from the fetal oral cavity was observed, with an irregular shape and soft texture, connected to the palate. The family agreed to send the fetal oral tumor for pathological examination. The induction procedure was successful, and the patient did not experience any significant discomfort. On the second day after induction, the patient’s blood pressure was 140/90 mmHg, and alanine aminotransferase (ALT) was 47 U/L. The patient recovered well after surgery.

**Figure 1**: Gross specimen of the stillborn fetus. A large tumor mass (T) measuring 16 × 15 × 8 cm protrudes from the oral cavity, with broad attachment to the palate (white arrowheads) and mandible (black arrowheads). The mass occupies the entire oropharyngeal space, consistent with the cause of upper airway obstruction and polyhydramnios observed prenatally. The irregular shape, solid-cystic consistency, and dark red appearance correlate with the heterogeneous imaging features and immature histology (Grade III).

### 3.2 Fetal specimen CT and MRI findings

#### 3.2.1 CT findings

**Figure 2:**
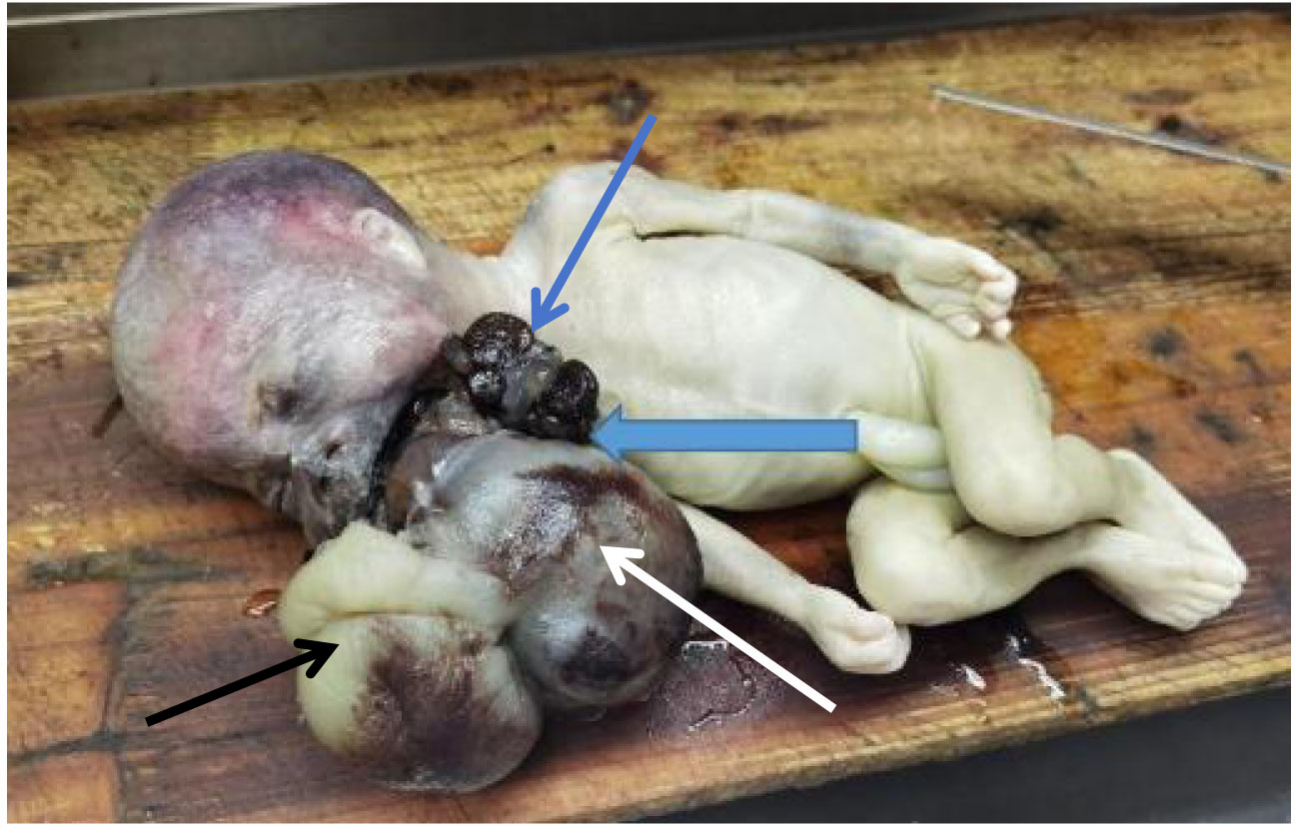
Gross specimen of the stillborn fetus. A large tumor mass (red arrowheads) measuring 16 × 15 × 8 cm protrudes from the oral cavity (blue arrowheads), with broad attachment to the palate (white arrowheads) and mandible (black arrowheads). The mass occupies the entire oropharyngeal space, consistent with the cause of upper airway obstruction and polyhydramnios observed prenatally. The irregular shape, solid-cystic consistency, and dark red appearance correlate with the heterogeneous imaging features and immature histology (Grade III). The solid-cystic appearance correlates with the heterogeneous density on CT and mixed signal intensity on MRI (**Figures 1**, **3**).

A large mass of soft tissue was observed within the fetal oral cavity, measuring approximately 6.1 × 6.1 × 6.8 cm. It partially protruded externally from the oral cavity, with clear boundaries and a visible capsule. The density of the soft tissue mass was uneven, showing cystic and solid changes. The solid portion had homogeneous soft-tissue density, while the cystic portion had lower density and exhibited punctate calcifications within the solid component—a feature more commonly associated with immature teratomas. The lesion was attached to the skull base, palate, oropharynx, and mandible with a broad base. Erosion and destruction of the mandibular bone were visible (**Figure 2**), reflecting the locally invasive behavior characteristic of high-grade immature teratoma. Vascularity could not be assessed on non-contrast CT.

**Figure 3:**
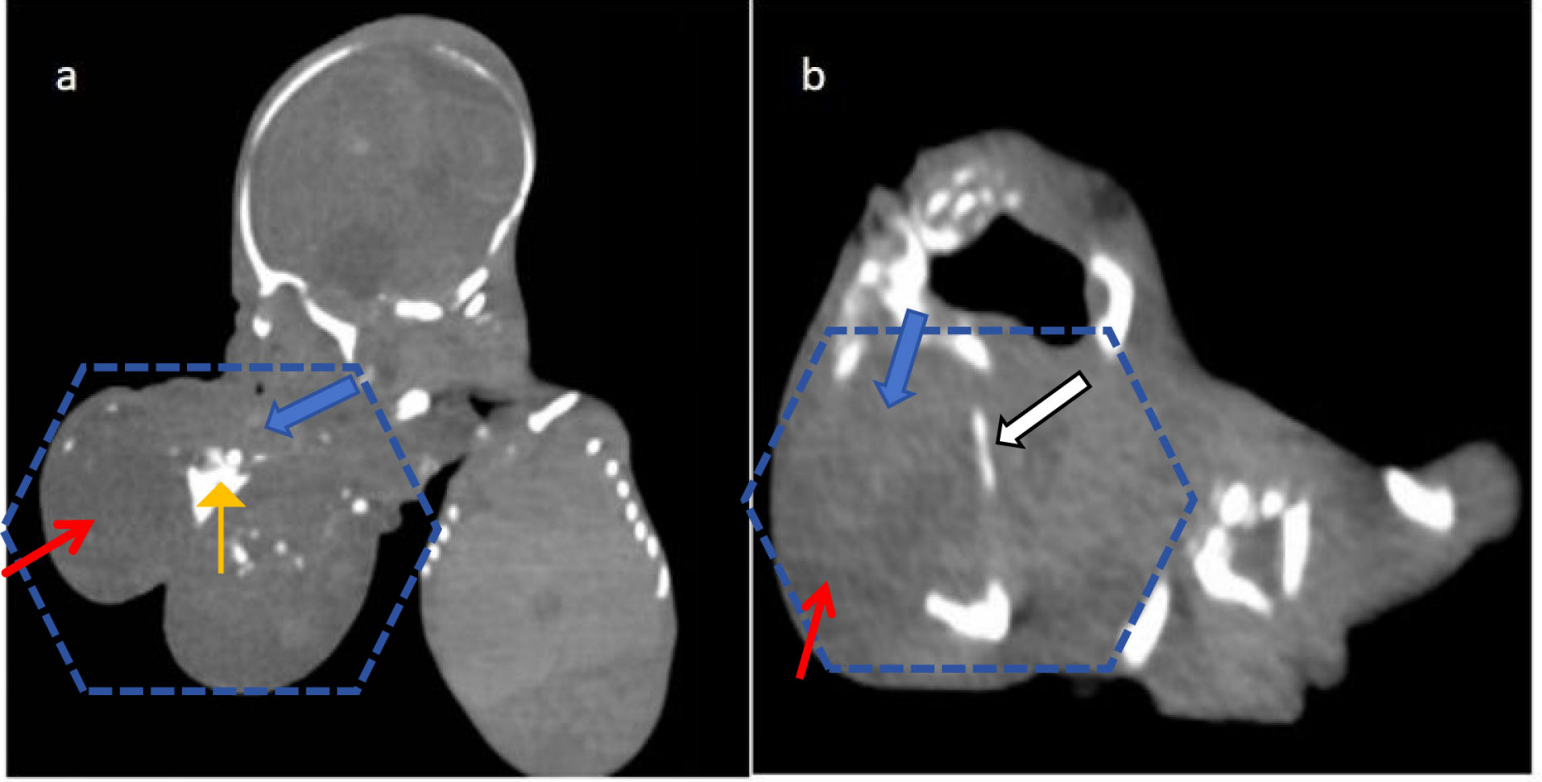
Postnatal CT findings of fetal oral immature teratoma. **(a)** Sagittal view shows a large mass (dashed outline) measuring 6.1 × 6.1 × 6.8 cm within the oral cavity, with broad-based attachment to the skull base, palate, and mandible. The tumor demonstrates heterogeneous density with mixed solid (S, blue arrows) and cystic (C, red arrows) components. Punctate calcifications (orange arrows) are visible within the solid portion, a feature more commonly associated with immature teratomas compared to mature counterparts. The mass causes significant oropharyngeal narrowing, indicating airway compromise. Vascularity: Non-contrast CT cannot directly assess tumor blood supply; however, the solid component shows homogeneous soft-tissue density without visible intratumoral vessels, a pattern consistent with moderately vascular immature teratomas. **(b)** Transverse view reveals erosion and destruction of the mandibular bone (white arrows), reflecting the locally invasive behavior characteristic of high-grade immature teratoma. The tumor mass is outlined by a dashed contour, with mixed solid (S, blue arrows) and cystic (C, red arrows) components. S, solid component; C, cystic component.

#### 3.2.2 MRI findings

A solid-cystic mass protruding from the fetal facial region was observed, measuring approximately 6.1 × 6.1 × 6.8 cm. The solid portion showed intermediate signal intensity on T1-weighted imaging (T1WI) and T2-weighted imaging (T2WI), while the cystic portion appeared as low signal intensity on T1WI and high signal intensity on T2WI. The lesion was attached to the skull base, palate, oropharynx, and mandible with a broad base, most of which protruded externally from the oral cavity, causing severe oropharyngeal compression and correlating with the risk of upper airway obstruction. On the right temporal lobe of the fetal brain, a well-defined T2WI hyperintensity, measuring approximately 1.7 × 1.7 cm, was visible, causing compression on the adjacent temporal lobe, compatible with an arachnoid cyst (**Figure 3**). The coexistence of an intracranial cystic lesion with an oral teratoma raises consideration of a developmental field defect involving midline structures. Postnatal MRI after induced delivery (**Figures 3C, D**) confirmed the broad-based attachment of the residual mass to the skull base and mandible with associated bony erosion, and additionally revealed a right temporal meningocele, further supporting the presence of concurrent neurodevelopmental anomalies.

### 3.3 Macroscopic examination

**Figure 4:**
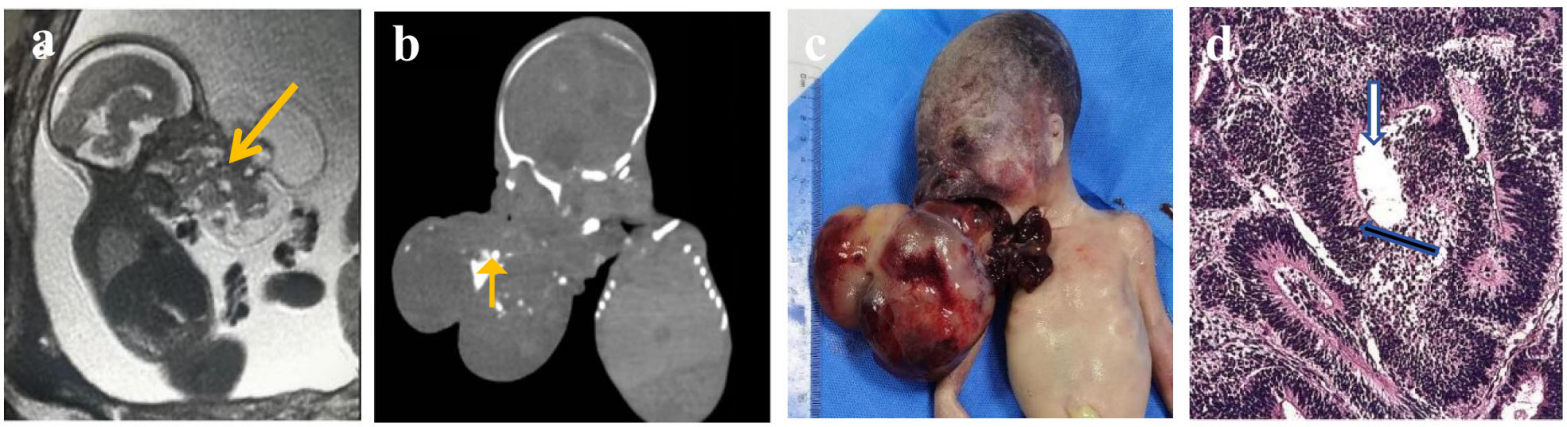
Gross section of the tumor specimen. The cut surface reveals an irregular solid-cystic structure measuring 8 × 8 × 7 cm. The solid areas (S, white arrows) appear firm and whitish, correlating with the solid components seen on CT and MRI. The cystic cavities (C, black arrows) contain dark red fluid (blue arrows), corresponding to the low-density and high-T2-signal areas on imaging. This macroscopic appearance is characteristic of immature teratomas, in which heterogeneous tissues derived from all three germ layers are present. S, solid component; C, cystic component.

General examination (**Figure 4**): The fetus measured 25 cm × 12 cm × 10 cm. There was a protruding mass in the oral cavity, measuring approximately 8 × 8 × 7 cm. The mass appeared dark red and had an irregular solid-cystic structure. The cystic component contained dark red fluid. The mass was connected to the palate and skull base, and some areas showed firm consistency. The solid areas correlated with the solid components seen on CT and MRI, while the cystic cavities corresponded to the low-density and high-T2-signal areas on imaging. No definite organ formation was observed.

### 3.4 Histopathological examination

Microscopic observation (**Supplementary Figure 1**): The mass consisted of three embryonic tissue layers. Skin appendages, neural glial tissue, choroid plexus, retina, cartilage, smooth muscle, adipose tissue, and vascular tissue were observed. Immature components included primitive neuroepithelium, immature neural glia, immature cartilage, and primitive stromal components, among others. These histopathological features—particularly the primitive neuroepithelium and immature cartilage—correspond to the solid components with calcification observed on CT and the restricted diffusion (high DWI signal) noted on prenatal MRI.

### 3.5 Pathological diagnosis

Immature teratoma in the fetus, Grade III. (Grade III diagnostic criteria: Minimal or absent mature tissue; abundant neuroepithelial component, along with a cellular stroma occupying four or more low-power fields in a single slide).The direct imaging-pathology correlation is summarized in **Supplementary Figure 1**, which aligns the calcifications and solid components on imaging with the immature neuroepithelium and primitive cartilage on histopathology.

### 3.6 Imaging-pathology correlation

As demonstrated in **Supplementary Figure 1**, the punctate calcifications observed on CT (**Supplementary Figure 1B**) and the low-signal foci on MRI (**Supplementary Figure 1A**) correspond histopathologically to immature neuroepithelial rosettes and primitive mesenchymal tissue (**Supplementary Figure 1D**). The solid components on imaging correlate with the solid areas of the gross specimen (**Supplementary Figure 1C**). This correlation confirms the Grade III immature teratoma and highlights the value of multimodal imaging in predicting histologic grade.

### 2.7 Follow-up results

Follow-up at 6 months showed that the mother’s condition was good.

## 4 Discussion

A teratoma is a tumor derived from germ cells or pluripotent cells of the embryo, primarily occurring in midline axial organs (1), such as the sacrococcygeal region, intracranial region, and sublingual region. Among these, sacrococcygeal teratoma is the most common, with an incidence rate of 1/40,000 to 1/20,000 (2). Approximately 6%-10% of teratomas are head and neck teratomas, most commonly located in the neck, while those originating from the oral cavity and connected to the palate and skull base are even rarer. Huang Ying et al. (4) reported a case in which ultrasound misdiagnosed the teratoma as conjoined twin malformation, but it was confirmed to be an oral maxillary teratoma after induced labor. Lai Qiurong et al. (5) and Chen Huiying (6) each reported a case of fetal oral teratoma, both originating from the tongue. More recently, Anjum et al. (7) reported a case of large oral immature teratoma in a 20-week fetus, measuring 10.8 × 6.7 cm, with histological confirmation of immature components. This case similarly resulted in termination and lacked intracranial abnormalities, further highlighting the rarity of concurrent intracranial involvement observed in our case.

To systematically review prior cases, a literature summary is presented in **Table 1**. Among the reported cases, only 25% utilized fetal MRI, and none provided direct imaging-pathology correlation. Intracranial abnormalities were absent in all prior cases, highlighting the novelty of the present case, which demonstrates concurrent arachnoid cyst and meningocele.

**Table 1:** Summary of reported cases of fetal oral teratoma with imaging and pathological correlation.

Author, year	Gestational age	Tumor location	Imaging modalities	Tumor size (cm)	Solid/cystic	Calcification	Intracranial abnormality	Histological grade	Perinatal outcome	Ref.
Sarioglu et al., 2003	24 weeks	Oral cavity (epignathus)	US, MRI	11.9 × 7.7 × 9.3	Mixed	Yes (bone formation)	None	Not reported	Termination	(3)
Huang et al., 2005	24 weeks	Palate	US	10.3 × 9.8	Mixed	NR	None	Mature	Termination	(4)
Lai et al., 2009	37 + 4 weeks	Tongue	US	12.5 × 11.8 × 8.9	Mixed	Yes	None	Mature	Termination	(5)
Chen et al., 2006	28 weeks	Tongue	US	6.2 × 5.2	Mixed (solid-predominant)	NR	None	Immature	Termination	(6)
Anjum et al., 2024	20 weeks	Oropharynx (soft palate)	US	10.8 × 6.7	Mixed (with cystic spaces)	NR	None	Immature	Termination	(7)
Present case	23 weeks	Oral cavity	US, MRI, CT	6.1 × 6.8	Mixed	Yes	Arachnoid cyst, meningocele	Immature (Grade III)	Termination	—

US, ultrasound; MRI, magnetic resonance imaging; CT, computed tomography; NR, not reported.

### 4.1 Pattern analysis

Among the 5 previously reported cases (including Anjum et al., 2024), only 2 (40%) utilized fetal MRI, and none provided direct imaging-pathology correlation. Intracranial abnormalities were absent in all prior cases, highlighting the novelty of the present case, which demonstrates concurrent arachnoid cyst and meningocele. Anjum et al. (7) reported a 20-week fetus with a large oral immature teratoma (10.8 × 6.7 cm) without intracranial involvement, further emphasizing the uniqueness of our case. The present case is also the only one to employ postnatal CT for evaluating bone destruction and to integrate multimodal imaging (US, MRI, CT) with histopathology.

### 4.2 Knowledge gaps

Limited use of MRI, lack of standardized imaging descriptors, and absence of histologic-imaging correlation in prior literature underscore the novelty of this report.

Fetal teratomas are classified as mature and immature. The proportion of immature teratomas is not accurately known. In cases of prenatal diagnosis of teratomas, it has been reported that 61% were diagnosed as mature teratomas, 39% as immature teratomas, and no cases as malignant teratomas (8). Sacrococcygeal teratomas are mostly benign, while the occurrence of teratomas in other locations is rare, with varying reported proportions. Yoneda et al. (8) reported proportions of 61% and 39% in prenatal diagnosed cases. Liu Xinyou et al. (9) reported that all 12 cases of fetal teratomas were benign. Heerema, McKenney, et al. (10) divided cases based on tumor surgery *in utero*, tumor discovered *in utero* but operated on post-birth, and tumor discovered and operated on post-birth into three groups. Group 1 consisted of 100% immature teratomas, group 2 consisted of 71% immature teratomas, and group 3 consisted of 30% immature teratomas.

Immature teratomas often exhibit invasive growth, which can damage surrounding tissues and organs, leading to fetal death (8). Imaging features that distinguish immature from mature teratomas include the presence of calcifications within solid components, invasive bone destruction, and restricted diffusion (high DWI signal) within solid areas—all of which were present in this case. These features correspond histopathologically to primitive neuroepithelium and immature cartilage. Fetal oral teratomas can block the oral cavity and result in excessive amniotic fluid, causing severe upper respiratory obstruction and high mortality rates (11, 12). Therefore, early detection is crucial. In this case, the immature teratoma originated from the fetal oral cavity, connected to the palate and skull base, protruding outward, exhibiting mobility, and forming a large mass that blocked the oral cavity. This led to upper airway obstruction and excessive amniotic fluid, which may have been the cause of intrauterine fetal death.

The embryological origin of teratomas is thought to involve pluripotent germ cells that undergo aberrant differentiation. The concurrent intracranial abnormalities in this case—an arachnoid cyst and a meningocele—may represent independent developmental anomalies rather than direct tumor extension. The mechanism underlying this association remains unclear; possibilities include a shared developmental field defect affecting midline structures, or a coincidental occurrence given the rarity of both conditions. No syndromic association was identified in this case.

Due to the complex composition of teratomas, their imaging characteristics vary, making diagnosis challenging. The pathological diagnosis in this case was an immature grade III teratoma, consisting of tissues from all three germ layers, including skin appendages, neuroglia, choroid plexus, retina, cartilage, smooth muscle, fat, blood vessels, and digestive tract tissue. Among them, the immature components included primitive neuroepithelium, immature neuroglia, immature cartilage, and primitive stromal components, among others. Compared to ultrasound examination, MRI provides a more macroscopic image with better tissue density display, enabling the identification of associated malformations, including those affecting the central nervous system, as well as the determination of airway compression (13). Combined with ultrasound and magnetic resonance imaging, it is now possible to achieve early prenatal diagnosis of fetal oral teratomas (12, 14, 15). In this case, prenatal MRI revealed that the tumor mass was connected to the palate and skull base, exhibiting a large size and protruding into the oral cavity, causing obvious obstruction of the oropharynx and laryngopharynx, and compressing and narrowing the trachea.

Literature reports indicate that approximately 18% of teratomas can be associated with other malformations, such as spina bifida and anencephaly (16). Therefore, it is important to also consider the development of other organs during diagnosis. In this case, MRI revealed unclear demarcation between the tumor mass and the skull base, along with the presence of an intracranial temporal arachnoid cyst and compression of the adjacent temporal lobe. Postnatal MRI additionally identified a right temporal meningocele, further supporting the presence of concurrent neurodevelopmental anomalies.

Differential diagnosis: When presented with a huge mass originating from the oral cavity, in addition to considering teratoma, one should also consider epignathus, dermoid cyst, and encephalocele. Epignathus typically arises from the palate with possible intracranial extension but lacks the solid-cystic heterogeneity and calcifications seen in teratomas. Dermoid cysts present as well-circumscribed unilocular cystic lesions without solid components or calcification. Encephalocele is characterized by a bony skull defect with continuous meningeal and brain tissue herniation, unlike the present case where the intracranial lesion was a separate arachnoid cyst without continuity. Cervical teratomas and lymphatic malformations should also be considered; the former lacks normal lip structure and is located within the oral cavity, while the latter present as cystic lesions with clear boundaries, rare occurrence of large masses, and no calcification or fat signals.

### 4.3 Prognosis and clinical management

Prenatal diagnosis of fetal oral teratomas has significant implications for counseling and delivery planning. The large tumor size (6.1 × 6.8 cm) and oropharyngeal obstruction in this case would have warranted consideration of an ex utero intrapartum treatment (EXIT) procedure if continuation of pregnancy had been pursued. EXIT allows for airway stabilization while maintaining fetomaternal circulation, which is critical in cases of anticipated airway compromise. Postnatal management includes surgical resection, with prognosis depending on tumor grade, resectability, and presence of associated anomalies. Studies have found that cystic teratomas have a better prognosis, while those with a higher tumor volume-to-fetal weight ratio have a poorer prognosis (17). Some studies have suggested termination of pregnancy for tumors with a diameter greater than 5 cm (18). Currently, induced labor is generally recommended in China. However, postmortem examination and pathological diagnosis after induced labor are extremely important, as they can confirm the presence of teratomas and determine their benign or malignant nature. In this case, postmortem examination revealed that the tumor affected the palate and skull base, and CT scans revealed destruction of the maxilla and mandible, suggesting a malignant immature teratoma.

### 4.4 Limitations

We acknowledge the limitations of this study. As a single case report, the findings may not be generalizable. Long-term follow-up of the mother is limited to 6 months, and no genetic or molecular analysis was performed on the tumor. Nevertheless, the detailed multimodal imaging and histopathologic correlation provide valuable insights for similar cases.

## 5 Conclusion

In conclusion, fetal oral teratomas are often large in size and can cause obstruction, fetal edema, or obstruct the birth canal during delivery. Therefore, early detection is of crucial importance in determining the fate of the fetus, guiding the mode of delivery, and determining the need for surgical treatment of the fetus and newborn. Multimodal imaging with systematic description of tumor location, size, composition, calcification, and mass effect enables accurate prenatal diagnosis and differentiation of immature from mature teratomas. RI provides a more macroscopic image with better tissue density display, allowing the identification of associated malformations and greatly assisting in the prenatal diagnosis of fetal oral teratomas. The presence of concurrent intracranial abnormalities, though rare, warrants thorough evaluation and may impact prenatal counseling and management planning.

## Data Availability

The original contributions presented in the study are included in the article/**Supplementary Material**. Further inquiries can be directed to the corresponding author.

## Supplementary material

Supplementary Figure 1 Imaging-pathology correlation of fetal oral immature teratoma (Grade III). **(A)** Prenatal sagittal T2-weighted MRI demonstrates a heterogeneous solid-cystic mass with multiple low-signal foci (orange arrows) within the solid component, suggesting calcification or immature tissue (e.g., primitive neuroepithelium). **(B)** Postnatal CT confirms the presence of punctate calcifications (orange arrows) within the solid portion, corresponding to the low-signal foci on MRI. **(C)** Gross specimen shows a dark-red, irregular mass with solid (S) and cystic (C) components. The solid areas correlate with the solid component on MRI (intermediate signal intensity) and the soft-tissue density on CT. **(D)** Histopathology (H&E, ×100) reveals immature neuroepithelial rosettes (black arrow) and primitive mesenchymal tissue (white arrow), which correspond to the solid components and calcifications seen on imaging. The abundance of immature neuroepithelium is consistent with Grade III immature teratoma (histologic grading criteria: immature neuroepithelium occupying ≥4 low-power fields per slide). These findings illustrate the imaging-pathology correlation of Grade III immature teratoma. The presence of calcifications on CT **(B)** and solid components on MRI **(A)** directly correspond to immature neuroepithelium and primitive cartilage on histopathology **(D)**, features that are absent in mature teratomas. Images in **(A)** and **(B)** are adapted from **Figures 1A**, **3A**, respectively, for direct comparison.

Supplementary Figure 2 Histopathological examination (H&E staining). Low-power view (×100) shows primitive neuroepithelial rosettes (black arrow) and cellular mesenchymal stroma (white arrow), diagnostic of immature teratoma. Immature cartilage and primitive neuroglial tissue are also present. The presence of abundant immature neuroepithelial components is consistent with a high-grade lesion. According to the grading criteria (abundant neuroepithelial component with cellular stroma occupying four or more low-power fields per slide), the tumor was classified as Grade III immature teratoma. These histopathological features—particularly the primitive neuroepithelium and immature cartilage—correspond to the solid components with calcification observed on CT and the restricted diffusion (high DWI signal) noted on prenatal MRI.
