# Supplementary figures and images for: A case report and literature review on imaging manifestations of immature teratoma in fetal oral cavity with concurrent intracranial abnormalities
Source: Front Oncol. 2026 May 8;16:1738161. doi: 10.3389/fonc.2026.1738161 (PMC13194022; doi:10.3389/fonc.2026.1738161)

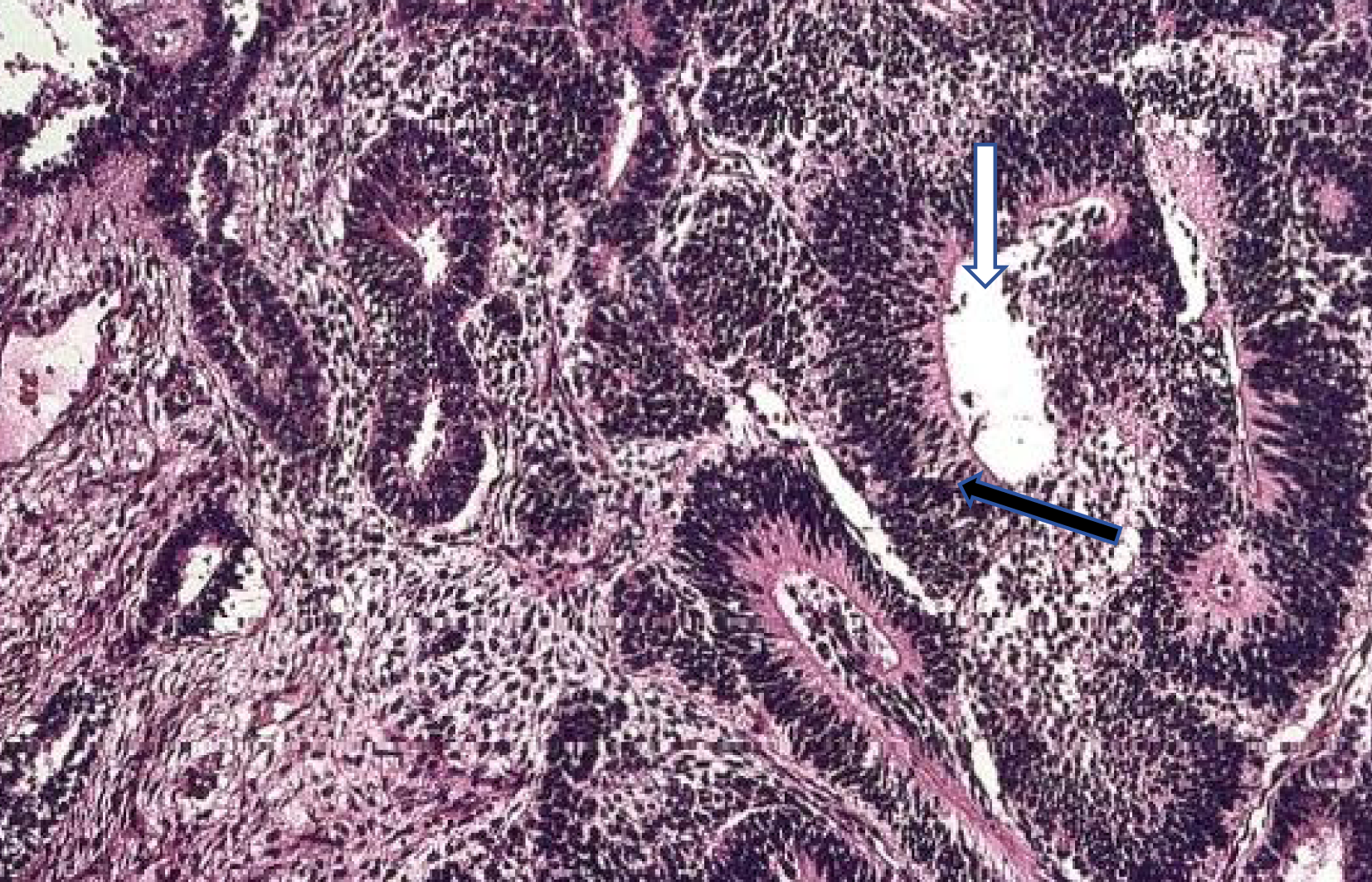

Supplement: Supplementary Figure 1 — Imaging-pathology correlation of fetal oral immature teratoma (Grade III). (A) Prenatal sagittal T2-weighted MRI demonstrates a heterogeneous solid-cystic mass with multiple low-signal foci (orange arrows) within the solid component, suggesting calcification or immature tissue (e.g., primitive neuroepithelium). (B) Postnatal CT confirms the presence of punctate calcifications (orange arrows) within the solid portion, corresponding to the low-signal foci on MRI. (C) Gross specimen shows a dark-red, irregular mass with solid (S) and cystic (C) components. The solid areas correlate with the solid component on MRI (intermediate signal intensity) and the soft-tissue density on CT. (D) Histopathology (H&E, ×100) reveals immature neuroepithelial rosettes (black arrow) and primitive mesenchymal tissue (white arrow), which correspond to the solid components and calcifications seen on imaging. The abundance of immature neuroepithelium is consistent with Grade III immature teratoma (histologic grading criteria: immature neuroepithelium occupying ≥4 low-power fields per slide). These findings illustrate the imaging-pathology correlation of Grade III immature teratoma. The presence of calcifications on CT (B) and solid components on MRI (A) directly correspond to immature neuroepithelium and primitive cartilage on histopathology (D), features that are absent in mature teratomas. Images in (A) and (B) are adapted from Figures 1A, 3A, respectively, for direct comparison. [file Image1.tif]

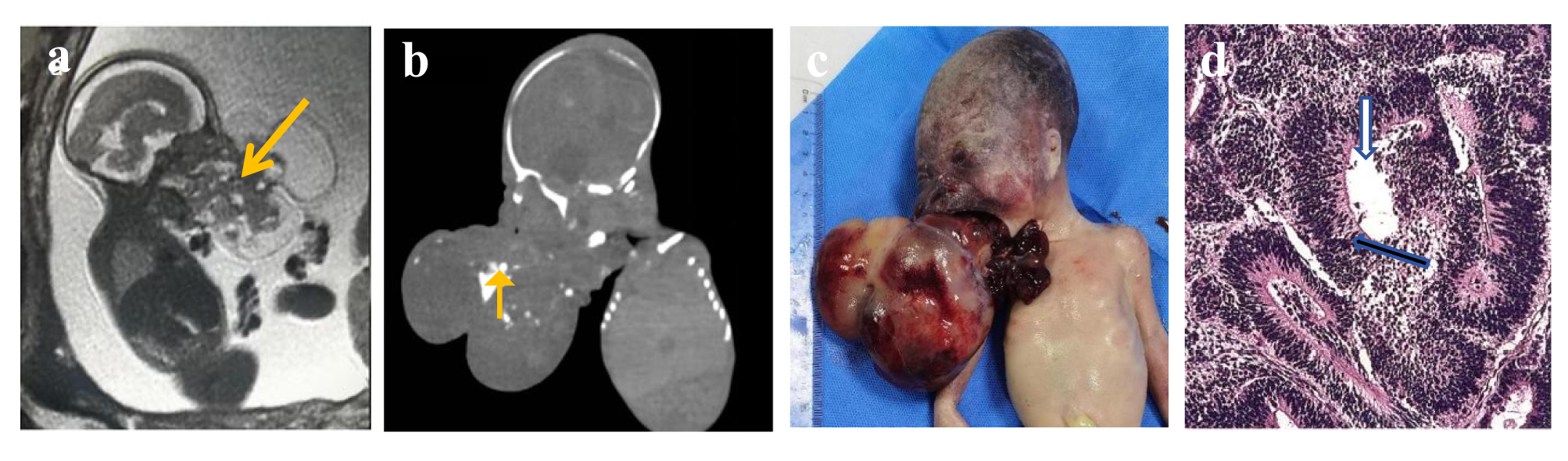

Supplement: Supplementary Figure 2 — Histopathological examination (H&E staining). Low-power view (×100) shows primitive neuroepithelial rosettes (black arrow) and cellular mesenchymal stroma (white arrow), diagnostic of immature teratoma. Immature cartilage and primitive neuroglial tissue are also present. The presence of abundant immature neuroepithelial components is consistent with a high-grade lesion. According to the grading criteria (abundant neuroepithelial component with cellular stroma occupying four or more low-power fields per slide), the tumor was classified as Grade III immature teratoma. These histopathological features—particularly the primitive neuroepithelium and immature cartilage—correspond to the solid components with calcification observed on CT and the restricted diffusion (high DWI signal) noted on prenatal MRI. [file Image2.tif]
